# The Clinical Conundrum of Cyclic Vomiting in the Cannabinoid User: Simply Cannabinoid Hyperemesis or Could It Be More?

**DOI:** 10.14309/crj.0000000000001711

**Published:** 2025-05-21

**Authors:** Kenan Sinan, Serina Beydoun, Tony Lulgjuraj

**Affiliations:** 1 Wayne State University School of Medicine, Detroit, MI; 2 Division of Pediatric Gastroenterology, Children's Hospital of Michigan, Detroit, MI; 3 College of Medicine, Central Michigan University, Mount Pleasent, MI

**Keywords:** cyclic vomiting syndrome, catamenial, adolescent, cannabinoid hyperemesis

## Abstract

Distinguishing between cannabinoid hyperemesis syndrome and cyclic vomiting syndrome (CVS) in patients with cyclical vomiting and heavy cannabinoid use is a significant diagnostic challenge. A critical consideration is catamenial CVS, a subtype of CVS in which vomiting episodes are closely linked to the menstrual cycle. This pattern is frequently overlooked in cannabinoid users due to the overlapping symptomatology of both conditions. However, identifying a menstrual association with vomiting episodes is crucial for diagnosing catamenial CVS because hormonal contraceptive therapy is an effective treatment. We present 2 adolescent cases of cyclical vomiting with cannabis use, where further clinical investigation revealed the menstrual cycle as a key trigger for vomiting, ultimately leading to successful treatment with hormonal contraception despite continued cannabis use. Clinicians should always consider menstrual history in patients with cyclical vomiting to facilitate early and accurate diagnosis and timely treatment of catamenial CVS.

## INTRODUCTION

Encountering cannabinoid use in patients with vomiting presents a challenging diagnostic scenario for clinicians. It often raises the question of whether they have cyclical vomiting syndrome (CVS) or cannabinoid hyperemesis syndrome (CHS). ROME IV criteria can diagnose both CVS and CHS. CVS criteria include stereotypical vomiting episodes of acute onset and absence of symptoms in between. CHS comprises of stereotypic vomiting episodes after prolonged cannabinoid use and symptom resolution after stopping cannabinoid use (Table 1).^1^ Hot showering, once a key feature of CHS, is now known to relieve symptoms in about 50% of patients with CVS.^2^ Distinguishing between these 2 diagnoses is crucial and poses a challenge for the clinician. Without a clear test to diagnose CHS, the best advice clinicians can offer their patients is unsatisfying: empiric cannabinoid abstinence for at least 3 vomiting cycles to differentiate CHS from CVS. This calls on the clinician to consider additional syndromes, such as catamenial CVS, with differentiating symptoms that when treated can lead to rapid clinical improvement.

**Table 1.** Comparing the characteristics of patients with cannabinoid hyperemesis syndrome and catamenial cyclic vomiting syndrome

	Cannabinoid hyperemesis syndrome	Catamenial cyclic vomiting syndrome
Cannabis use	+++++	++^a^
Periodicity	+++	+++++
Vomiting intensity	+++++	+++++
Response to hot showers	+++	+++
Response to capsaicin	+	+
Duration of episode	++	+
Relief of vomiting with cessation of cannabinoids	++++	No association

^a^ Indicates baseline population use.

Catamenial CVS is a well-defined subtype of CVS that is simple to identify when patients fit the clinical case definition. These patients will have typical CVS symptoms that occur predictably with the onset of menstruation and are generally asymptomatic between cycles.^3–5^ Vomiting cycles generally resolve quickly on initiation of hormonal contraception.^6,7^ We present 2 such cases of adolescent girls presenting with vomiting in the setting of heavy cannabis use who, on further history and clinical follow-up, were diagnosed with catamenial CVS.

## CASE REPORT

### Case 1

A 17-year-old girl presented to the emergency department (ED) due to 6 days of significant nausea, vomiting, and abdominal pain. She reported being on day 3 of her menstrual cycle; symptoms began before the start of menstrual flow. Medical evaluation was unremarkable. Capsaicin cream had been ineffective. Further history revealed ED visits for vomiting and dehydration correlating with onset of menstrual flow. Social history noted years-long, daily cannabis use. She remained hospitalized for 1 week and was discharged. With clinical features of both CHS and catamenial CVS, she was advised to both abstain from cannabis and start oral contraceptive pills (OCP).

Approximately 5 months later, she returned to the ED with similar symptoms. She reported initial improvement in symptoms with OCP use but was nonadherent due to forgetfulness and mood changes. She perceived no improvement in symptoms with cannabis abstinence for 3 months and resumed daily use. Her physician advised both resuming OCPs and abstaining from cannabis. At a 7-month follow-up, she had complete symptom resolution with consistent OCP use, despite ongoing, daily, heavy cannabis use.

### Case 2

A 15-year-old girl presented to the ED with a 4-day history of nonbloody, nonbilious vomiting. She reported 2 similar prior episodes, each with onset of her menstrual flow. She also reported years long, daily, heavy cannabis use. She was admitted for symptomatic management and discharged home with a diagnosis of CHS and possible catamenial CVS. She was both prescribed OCPs and advised to abstain from cannabis.

Approximately 1 month later, she returned to the ED with similar symptoms. She had not initiated OCPs and continued using cannabis. She was again treated symptomatically and discharged with OCPs and instructions to abstain from cannabis. On follow-up, she endorsed OCP adherence, without side effects, and reported no further episodes of vomiting, despite ongoing heavy, daily cannabis use.

## DISCUSSION

When evaluating pediatric patients for vomiting, clinicians must maintain a broad differential diagnosis including migraines, gastroparesis, metabolic disorders, and functional vomiting. These patients may undergo extensive testing—endoscopy, upper GI studies, biochemical testing, and cross-sectional imaging—to evaluate other causes of chronic vomiting. Ultimately, CVS and CHS are diagnosed clinically. The overlap in symptoms and lack of definitive tests make distinguishing the 2 challenging (Table 1). Further complicating matters, CHS is increasingly prevalent, likely due to rising recreational cannabinoid use and increasing product potency.^8,9^ Accurate assessment for CHS risk among adolescent patients is further complicated by underreporting of cannabinoid use and the difficulty these patients may have with the prolonged cannabinoid cessation required for diagnosis.^10^

Catamenial CVS is characterized by vomiting episodes that align with the menstrual cycle, often just before or at the onset of menstruation.^3–5^ Although limited, research indicates that 30%–50% of women with CVS have a catamenial pattern.^11,12^ These cases suggest that identifying an association between the onset of vomiting and onset of menses in women with cyclical vomiting allows the clinician to make a positive diagnosis of catamenial CVS and start highly effective hormonal contraception, even in women with heavy cannabinoid use.

The young women in these cases had multiple healthcare encounters before their catamenial CVS diagnosis, potentially reflecting the broader medical bias against considering menstrual-related disorders. Women have long faced underrepresentation, underdiagnosis, and undertreatment in medicine, a disparity that affects their healthcare quality and outcomes.^13^ These cases highlight that menstruating patients face unique clinical challenges and highlight how menstrual history and contraceptive options—including side effect profiles—should be part of individualized treatment planning.^14^ When side effects lead to OCP nonadherence, as in case 1, referral to gynecology may help explore the broad spectrum of hormonal contraceptives available.

Cyclical vomiting is a morbid problem with high direct and indirect costs. Misdiagnosing CHS based only on cyclical vomiting with cannabinoid use will lead to prolonged, unnecessary suffering when catamenial CVS is overlooked. A complete history—including the relationship between vomiting and menstruation—is critical. Proper identification of catamenial CVS can spare patients from unnecessary testing and diagnostic delay, and allow earlier treatment. Although these cases represent a small sample, they highlight that catamenial CVS can occur even in patients at high risk for CHS and that hormonal contraception can be effective despite ongoing heavy cannabinoid use. These findings, if verified in larger cohorts, may inform updates to diagnostic and treatment recommendations. In the interim, we recommend clinicians assess menstrual timing in all patients with cyclical vomiting and consider early OCP initiation when catamenial CVS is suspected, regardless of cannabinoid use history.

## DISCLOSURES

Author contributions: K. Sinan: writing and editing of the manuscript. S. Beydoun: writing and editing of the manuscript. T. Lulgjuarj: writing and editing of the manuscript, and is the article guarantor.

Financial disclosure: None to report.

Previous presentation: This case report was presented as a poster presentation at the North American Society of Gastroenterology, Hepatology, and Nutrition's Annual Meeting; November 7, 2024; Hollywood, Florida.

Informed consent was obtained for this case report.

Ethics statement: Informed consent was obtained from the patients discussed in this manuscript.
